# Digital Delivery of Cognitive Behavioral Therapy for Insomnia

**DOI:** 10.1007/s11920-019-1041-0

**Published:** 2019-06-04

**Authors:** Annemarie I. Luik, Tanja van der Zweerde, Annemieke van Straten, Jaap Lancee

**Affiliations:** 1 Department of Epidemiology, Erasmus MC University Medical Center, PO Box 20140, 3000 CA Rotterdam, Netherlands; 2 Department of Clinical, Neuro and Developmental Psychology, Amsterdam Public Health Research Institute, Vrije Universiteit Amsterdam, Amsterdam, Netherlands; 3 Department of Clinical Psychology, University of Amsterdam, Amsterdam, Netherlands; 4 PsyQ Amsterdam, Amsterdam, Netherlands

**Keywords:** Insomnia, Sleep, Cognitive behavioral therapy, CBT, Online, Internet

## Abstract

**Purpose of Review:**

Digital cognitive behavioral therapy (dCBT) has been available for over a decade. We reviewed the evidence that accumulated over the past 5 years and discuss the implications for introducing dCBT into standard healthcare.

**Recent Findings:**

Studies have consistently supported the use of dCBT to treat insomnia. Evidence is now demonstrating large short-term effects and smaller long-term effects up to 1.5 years after treatment across populations with various co-occurring health problems. The effects also extend into a range of psychological well-being factors. Mediators and moderators have been studied to understand mechanisms and create new opportunities to enhance effectiveness and reduce dropout. Incorporating personalized guidance in dCBT may further enhance effectiveness.

**Summary:**

The evidence for dCBT for insomnia is strong and suggests that dCBT is ready for application in standard healthcare. Further research, digital innovation, and development of effective implementation methods are required to ensure dCBT fulfills its potential.

## Introduction

Insomnia disorder is diagnosed when complaints of trouble falling asleep, staying asleep, or waking up too early occur three or more nights per week, for three or more months with significant daytime effects [1]. A substantial percentage of the population suffers from insomnia: estimates range up to 10% for insomnia disorder and 25% for insomnia symptoms [2].

Even though insomnia is a very prevalent disorder, only 37% of those suffering from insomnia report consulting a healthcare provider for their sleep problems [2]. Reasons for these low numbers of help-seeking behavior in persons with insomnia are likely varied, but a reason could be that insomnia disorder is often treated with medication [3, 4]. This is against American and European guidelines that recommend cognitive behavioral therapy (CBT) as the first-line treatment for insomnia disorder [5•, 6] because of its substantial clinical evidence base [7•]. CBT for insomnia is a structured therapy consisting of several components [8, 9]. The therapy typically includes educational, behavioral, and cognitive components. Often relaxation is also considered a standard component and other techniques such as mindfulness are added frequently. More detail about the components of CBT for insomnia can be found in Table 1.

**Table 1:** Components of cognitive behavioral therapy (CBT) for insomnia

CBT for insomnia component	Important techniques
Educational	Explaining the process and function of normal and disturbed sleep Explaining how behaviors can promote sleep, for example, by explaining the importance of a healthy lifestyle (e.g., low caffeine and alcohol intake) and by explaining the importance of bedtime routines (e.g., a dark bedroom and fixed bedtimes)
Behavioral	Stimulus control: advising a person to get out of bed when awake for more than 15 to 20 min, and only go back to bed when sleepy, to re-establish the connection between bed and sleep [10] Sleep restriction: restricting the time in bed to the average time slept in the past week (typically with a minimum of 5 h) to heighten the homeostatic sleep drive. If the sleep efficiency is above 85% or 90%, the amount of time in bed is lengthened [11]
Cognitive	Identifying and challenging misconceptions about sleep (such as “I have to sleep 8 h a night”) Addressing the “racing mind”: learning how to cope or deal with worries and thought that prevent the person from falling asleep Paradoxical intention: trying to stay awake instead of falling asleep [12]
Additive	Relaxation: relaxation and breathing techniques to teach persons to unwind [13] Mindfulness: using techniques from mindfulness to teach persons to unwind [14]

Yet, despite it being the recommended treatment, CBT for insomnia is hardly omnipresent. One of the most important factors for CBT not being widely available seems to be a shortage of therapists and resources. Digital CBT (dCBT) for insomnia was suggested more than a decade ago [15] as a potential solution to address this shortage: did it live up to its potential?

## What Is Digital CBT?

Before discussing dCBT for insomnia, specifically, it is important to focus on dCBT in general. Interest for the digital delivery of psychological therapies started in the 1990s. CBT turned out to be a particular suitable candidate because of its highly structured nature. This has led to a wealth of programs being developed and investigated, in particular for psychological problems such as depression and anxiety; generally, these programs have shown to be effective [16]. At first, digitalizing CBT happened by merely translating self-help manuals to a website. More elaborate programs that are highly personalized, tailored, and fully automated have become available since. In this manuscript, we will define dCBT as a “by digital means communicated form of CBT,” where digital covers the full spectrum of digital advances. This includes many other terms that have been used commonly to describe this format such as “online,” “electronic,” “mobile,” and “Internet.” To recognize that the future will likely bring techniques yet unknown, we choose to use the word “digital” here. The digital innovation underlying dCBT will have the capability to tailor the selection and sequencing of CBT elements to the individual, making dCBT highly adaptive. This fits with a personalized behavioral medicine approach [17], creating a reproducible, evidence-based therapy for anyone, anywhere, anytime.

Over the past decade, many dCBT programs have become available with very different levels of digitalization and automation, typically moving away from just publishing self-help information on the Internet. Three main ways have been recognized before [18] (see Table 2 for a short description and example programs for treating insomnia). Briefly, *supportive dCBT* is where dCBT is used mainly as a support function to augment face-to-face individual or group therapy, but the in-person therapy remains primary. Second is *therapist-guided dCBT*, where actual therapy content is communicated to the patient with the help of web pages or a mobile app containing text, images, videos, audio, or animations but is supported by a clinical professional. Third is *fully automated dCBT*, where the program is offered without any human support. Personalization is built into the automated program using algorithms to tailor all aspects of the program, including personalized feedback and a tailored treatment program.

**Table 2:** Automation of digital cognitive behavioral therapy (dCBT)

	Definition	Automation	Program examples
Supportive dCBT	dCBT elements are used to support face-to-face therapy	Limited	Gehrman et al. [19]
			Lichstein et al. [20]
			Kuhn et al. [21]
Therapist-guided dCBT	Automated dCBT with guidance and tailoring supported by human therapists	Partly	Lancee et al. [22]
			Van Straten et al. [23]
			Blom et al. [24]
Fully automated dCBT	Fully automated and tailored dCBT without in-person clinical support	Fully	Ritterband et al. [25]
			Vincent et al. [26]
			Espie et al. [27]

We will use the term dCBT for all these intervention formats, but it should be kept in mind that the literature comprises everything from optimized websites through to advanced algorithm-driven systems, each differing considerably in the amount of clinician time, level of automatization, costs, and scalability.

## Evidence for dCBT for Insomnia

Returning to dCBT for insomnia specifically, it was in 2004 when Ström and colleagues published the first randomized controlled study investigating an Internet-based treatment for insomnia [28]. They developed a guided program where dCBT was combined with human support. Since then, many more studies and digital programs for insomnia have been developed, both integrating human support [22–24, 29–31] and fully automated [25–27, 32–34] dCBT. The number of online treatments for insomnia has been growing rapidly and will likely continue to do so. It is important to realize that only a small percentage of programs have been studied in formal research studies, leaving many more programs without clinical evidence available. As scientists in this field, we should emphasize the need for clinical evidence for these programs as they are offered to a vulnerable population, potentially accompanied by unknown efficacy and risks, with the possibility of even causing harm.

Overall, evidence has consistently been demonstrating that dCBT for insomnia is efficacious in treating insomnia. Meta-analyses [35•, 36] demonstrate sizeable effects of dCBT on insomnia. Narrative reviews published over the past years have addressed and identified multiple gaps in the evidence base [18, 37], and considerable amounts of new evidence have been published since. Recent collected evidence mainly centers around four themes: (1) short- and long-term efficacy across populations, (2) effects of dCBT beyond insomnia, 3) improvement of dCBT effectiveness: attrition, non-response, moderators, and mediators; and (4) the role of human support in dCBT.

### Evidence for Short- and Long-term Efficacy Across Populations

Since the 2016 meta-analyses of Zachariae et al. [35•] and Seyffert et al. [36], a large number of randomized controlled trials investigating existing and new dCBT for insomnia programs have been published [31, 33, 34, 38–59]. These studies reliably show positive effects on insomnia, and most report large effects with Cohen’s *d* > 1.0, which is similar or larger to effects reported in the previous meta-analyses on dCBT for insomnia. Additionally, the evidence for long-term effects of dCBT has increased, with trials showing sustained effects in uncontrolled follow-ups up to 1.5 years after randomization [60–62]. One of these studies [62] also assessed the effect in comparison with a control group after 1 year; no significant difference was observed, probably due to improvement of insomnia complaints in the control group. A study assessing a 3-year follow-up period did not find sustained effects for a reduction in insomnia severity, but this study did show that less sleep medication was used by participants who had received dCBT and that the control group had received more additional insomnia treatments during the follow-up period [63].

The positive effects of dCBT on insomnia symptoms are not only seen in adults with insomnia solely. These effects were also established in adolescents [31, 64], breast cancer survivors [65], and patients with concurrent disorders such as depression [38, 57], tinnitus [45], asthma [54], or heightened blood pressure [48] for example. This suggests that dCBT can be of use in a range of populations, even when the target population has insomnia concurrent with other disorders.

### Evidence for Effects of dCBT for Insomnia Beyond Insomnia

Much of the evidence collected in the past years has focused on the improvement of symptoms of diseases that are often comorbid with insomnia, in particular symptoms of other mental health disorders [66]. First and foremost, a wealth of evidence has demonstrated that dCBT for insomnia is reducing depressive symptomatology. This has been demonstrated by using dCBT for insomnia not only as an adjunct therapy [57], but also as stand-alone treatment in trials including participants with comorbid symptoms of insomnia and depression [41, 55, 59], independent of whether the treatment was fully automated or guided by healthcare professionals. The effects sizes are typically in the moderate-to-large range. Additionally, in audits of clinical services, improvements in depression were equally seen [67, 68], suggesting these effects hold in real-world, clinical settings. It has even been suggested that the effect of dCBT for insomnia is in the same range as CBT specifically aimed at treating depression [59]. This was confirmed by a direct comparison which demonstrated a within-group post-treatment effect of dCBT for insomnia on depressive symptoms (Cohen’s *d* 0.74) similar to that of dCBT for depression on depressive symptoms (Cohen’s *d* 0.66) [38]. The effects have been suggested to retain over time [38, 69], although this was not confirmed by a study with an uncontrolled follow-up [53]. Positive effects of dCBT for insomnia were also seen on symptoms of other mental health disorders, for example, a small-to-moderate effect on anxiety [52•, 68, 70, 71] and a small effect on psychotic experiences and hallucinations [47]. Two randomized controlled trials specifically designed to test mediation have now also demonstrated that a substantial proportion of the improvements in mental health symptoms are mediated by the improvement in insomnia symptoms [47, 52•]. It is important to note that most of the studies mentioned above have been carried out in subclinical samples.

In addition to improvements in mental health, some evidence has emerged that dCBT for insomnia is also effective in reducing cognitive complaints [52•] and improving cognitive performance when assessed with neuropsychological tests [72]; although the effect sizes are typically small. Potentially related to this, self-rated work productivity also shows a small-to-moderate increase after dCBT, together with other work-related constructs such as job satisfaction and work-related rumination [39, 40, 46].

### Improving dCBT Effectiveness: Attrition, Non-response, Moderators, and Mediators

Although dCBT is generally effective in improving insomnia, large steps can still be taken to improve the effectiveness and decrease attrition. Several approaches have been taken to gain more insight in these topics over the past few years.

First, average treatment adherence is around 50% for dCBT for insomnia; attrition and adherence therefore need our attention [73]. This is particularly true for studies with no telephone or face-to-face contact, which might be more similar to implementations in some large-scale, real-world scenarios. This is however not unique to dCBT for insomnia; dCBT programs for other disorders have equal, or even larger, problems with attrition [74]. So far, it has proven difficult to find indicators of dropout; studies have suggested that a lower social economic status [55], a longer sleep duration, less severe insomnia complaints, and more depressive symptoms increase dropout [75].

Second, moderators of treatment effects have equally proven hard to establish. For example, no moderating effect of sex, age, race, education, or income on treatment outcome was established [55]. Insomnia-specific indicators such as acceptance of diagnosis-related problems as well as negative emotions and cognitions were associated with better treatment results [76].

Third, knowing by which factors the improvement in insomnia is mediated might help us gain insight in mechanisms underlying dCBT and potentially help us further increase effectiveness. Several cognitive processes have been hypothesized to mediate insomnia improvement. In the past 5 years, the evidence for a role of dysfunctional beliefs about sleep as a mediating factor in the improvement of insomnia after dCBT has grown substantially [30, 51, 58]. Additionally, knowledge, locus of control, sleep-related worry, and pre-sleep arousal have been suggested to mediate improvement in sleep [51, 58].

### dCBT: Do We Need a Human Therapist?

Substantial effects of programs both with and without human guidance have been found [7•, 35•, 36], but formal comparisons are scarce. More support, whether via a human therapist or automated, is generally thought to have positive effects on adherence and efficacy of dCBT for depression and anxiety [77]. A study comparing dCBT for insomnia with and without additional motivational support via email showed increased effects for the condition with support [78]. A study assessing human support in a dCBT for insomnia program for adolescents identified four factors that were part of support: forging a working alliance, forging therapy integrity, and forging a positive attitude and sleep expertise. Of these four, only sleep expertise seemed to contribute to improvements [79].

Of note, human support could also take place outside the sphere of mental healthcare professionals; support via web fora was experienced as positive by those taking a dCBT course [80] for example. Research studies making a valid comparison of automated support with no or human support are lacking, but automated support in the form of reminders has been demonstrated to enhance adherence [81]. If dCBT does not reach the same effectiveness without guidance, this does not reduce the value of dCBT per se. It still creates a tremendous opportunity to offer dCBT to those with less complicated complaints in a stepped care approach, as has been previously suggested [15]. A recent study assessed this by increasing the amount of therapist guidance based on the treatment response in the first weeks of dCBT. By increasing guidance, the treatment effect was increased and the number of failed treatments reduced [56•].

Another question often asked is how dCBT compares to face-to-face therapy. Meta-analyses have suggested effect sizes of dCBT for other disorders are in a similar range as face-to-face CBT [82]. Similarly, this has been suggested by comparing effect sizes of meta-analyses incorporating dCBT for insomnia programs with meta-analyses analyzing face-to-face CBT programs [7•, 35•, 36]. However, direct comparisons are still scarce. One study directly comparing face-to-face CBT with dCBT demonstrated that face-to-face CBT outperformed dCBT [43•]. A later study in military personnel confirmed this [50]. A comparison of dCBT against group CBT did however not show any significant differences in improvement in insomnia [24].

Although it might very well be that human involvement in treatment increases effectiveness, we have to note that most dCBT programs so far do not take full advantage of the possibilities that digital solutions can offer to personalize feedback, tailor treatments, and create a therapy bond. It could be that it might not be the support of a human per se, but rather individual tailoring and attention that is crucial to the effectiveness.

## The Future of Digital Treatments for Insomnia

Although evidence has been mounting in the last 5 years, this has only resulted in a relatively small increase in the use of dCBT for insomnia in clinical services. The use of dCBT for insomnia seems to remain mostly ad hoc and limited as was noted a decade ago [15]. New initiatives have been seen, for example, by companies providing dCBT approaches for employees in the USA (e.g., www.bighealth.com/sleepio), the UK National Health Service adopting dCBT for insomnia in some of their standard care facilities (e.g., www.good-thinking.uk), and initiatives to integrate dCBT into Dutch GP practices [83]. A consistent overall approach is still lacking, not at least due to different healthcare systems across countries, a lack of knowledge about dCBT for insomnia among professionals and patients, and missing quality control standards for dCBT programs. The role that dCBT for insomnia can play in general healthcare therefore remains unclear for many healthcare professionals. Fulfilling the full potential of dCBT for insomnia is thus a process that still needs work, with many challenges in the field of science and implementation.

### The Research Gaps

Despite the fact that the evidence for the positive impact of dCBT for insomnia is strong, several research gaps still exist in the evidence base. We highlight three areas in this paper: (1) negative side effects and harm, (2) insights in moderators and mediators, and (3) costs and savings.

First, negative side effects and potential harm of CBT have in general received little attention when studying CBT; for dCBT, this has not been any different. Recently, one RCT reported side effects of dCBT for insomnia. Participants that received dCBT reported a higher occurrence of headaches, fatigue, extreme sleepiness, reduced energy, irritability, difficulty remembering, and difficulty concentrating than the waitlist control group [52•]. Although based on other outcomes of this trial there is a reason to believe these side effects may be short-lived, they do deserve more of our attention, particularly as they may play a role in attrition. Indeed, we argue that assessing side effects should become standard in each trial on dCBT, irrespective of the disorder investigated.

Second, we need to improve our understanding of moderators and mediators of the effects of dCBT for insomnia to be able to understand the mechanisms at work. This can also further improve treatment and reduce attrition. Although several studies have increased our understanding [30, 47, 51, 52•, 55, 58, 75, 76], we still have a limited understanding of why people drop out and why some do not respond. This is also true for in-person CBT; we still do not know why a third of patients do not respond to CBT for insomnia [84]. dCBT can play a crucial role in understanding these mediators and moderators as it facilitates doing large-scale trials with standardized treatments, ensuring the statistical power to study these associations. This also includes increasing attention for factors known to affect adherence from the broader digital interventions literature, for example, the use of persuasive technology elements [85].

Third, if we want to make dCBT for insomnia a success and ensure implementation in healthcare, we have to demonstrate the costs and savings that accompany dCBT. Some studies assessing costs have appeared over the past years. A cost-benefit analysis of applying dCBT for insomnia in a working population has been estimated to lead to a net benefit of USD 512 per participant [44]. A comparison of dCBT and group CBT for insomnia in adolescents has suggested that dCBT outperforms group CBT with similar results but at lower costs [42]. To really persuade the world dCBT for insomnia deserves a place, we will need more of these studies generating convincing evidence on cost-effectiveness.

Lastly, we would like to end this paragraph with emphasizing that clinical evidence for one program does not mean clinical evidence for all programs. We cannot simply assume that every program that contains CBT is effective simply because it contains evidence-based CBT elements.

### Advantages and Disadvantages for Implementing dCBT in Routine Healthcare

The main advantage of dCBT is the ability to reach many more persons with insomnia complaints than can ever be reached with face-to-face CBT, especially in a fully automated format. It is simply impossible to sufficiently train enough therapists to be able to offer face-to-face CBT for insomnia to everyone in need, because of high prevalence rates of insomnia and limited healthcare budgets. Additionally, dCBT has the advantage of being able to reach those who cannot take therapy sessions during working hours as it is available 24/7, those in remote locations as it is available anywhere with a sufficient Internet connection, and those not wanting to visit a mental healthcare clinic as it can be available from the convenience of your own home.

Naturally, dCBT does not only have advantages. One of the main disadvantages is that there is less clinical control over the treatment process than in face-to-face settings, which can pose problems for certain mental health complaints, most prominently suicidality. Therefore, dCBT will need rigorous methods to ensure it can be acted upon when these complaints are endorsed, in both guided and fully automated programs. So far, programs have offered contact details of healthcare professionals or services such as local suicide prevention lifelines, but potentially more can be done to help this group. However, we do need to realize that this is not that dissimilar to face-to-face CBT, as a therapist will not be available 24/7 either. Currently, the regulation of dCBT is very limited and largely dependent on the standards companies or institutions set themselves. A more regulated approach is urgently needed to ensure that dCBT causes benefit and not harm. It is important to keep in mind that the absence of an effect can also be harmful when it limits motivation or faith in future or other treatment opportunities. Next, while we live in a world that becomes more and more digitalized, there will still be those who prefer or need in-person treatment. The use of dCBT can however help to free up time from healthcare professionals to treat those that need or want in-person treatment. Lastly, it is important to realize that digitalization is not a completed process. New digital techniques appear constantly. This can lead to dCBT programs quickly being becoming outdated. Therefore, dCBT will have to continue changing, not just to keep up with the newest technologies, but because these technologies can help improve dCBT. This however creates an additional challenge for regulating bodies as the collection of scientific evidence is typically much slower than any digital advance.

## Conclusions

The clinical evidence for dCBT for insomnia has substantially increased over the last few years. Additional evidence has emerged around the short- and long-term efficacy across populations; the effects of dCBT beyond insomnia, attrition, moderators, and mediators of dCBT; and the role of human support in dCBT. The field is by no means done however: important research gaps exist around negative side effects and potential harm, insights in moderators and mediators, and costs and savings. However, we think that dCBT for insomnia is ready for a wider application in healthcare. It will help us ensuring that those in need of help for insomnia can receive the recommended treatment.
